# Short-Term Pesticide Exposure Reshapes Soil Fungal Communities in a Soil-Dependent Manner

**DOI:** 10.3390/jof12080584

**Published:** 2026-08-07

**Authors:** Veronika Řezáčová, Oushadee A. J. Abeyawardana, Milan Řezáč, Ema Némethová

**Affiliations:** Czech Agrifood Research Center, Drnovská 507, Ruzyně, 161 00 Prague, Czech Republic; veronika.rezacova@carc.cz (V.Ř.);

**Keywords:** soil fungi, pesticides, short-term exposure, soil pH, trophic groups

## Abstract

Soil fungi play key roles in decomposition, nutrient cycling, plant–soil interactions, and soil structure, yet their immediate responses to pesticides remain insufficiently understood. Most studies have focused on long-term, cumulative, or plant-mediated effects, limiting insight into direct impacts. We assessed the short-term direct effects of eight commercial pesticides on fungal abundance, alpha diversity, community composition, trophic structure, and total arbuscular mycorrhizal fungal (AMF) abundance across three contrasting agricultural soils in a plant-free pot experiment. Responses varied strongly among soils and pesticides, with no consistent fungal suppression. Alpha-diversity changed little and inconsistently, whereas community composition shifted markedly, indicating rapid community reorganization before detectable changes in richness or evenness. Soil 1 showed the strongest responses, while Soil 2 was resistant. Although most trophic groups’ relative abundance remained stable, their internal composition was often substantially restructured, suggesting taxonomic turnover without major functional-group shifts. Total AMF abundance increased under several treatments in Soil 1 but decreased under selected treatments in Soil 3. Soil pH was associated with fungal diversity, community composition, and total AMF abundance; however, pesticide-induced pH changes did not consistently explain microbial responses. Overall, short-term pesticide exposure drove selective, soil-dependent community restructuring rather than uniform diversity loss, supporting soil-specific risk assessment.

## 1. Introduction

Pesticide application has become a common practice in agriculture to control weeds, pests, and diseases, thereby increasing quality and productivity. However, they can negatively impact non-targeted organisms, including microorganisms [1], leading to imbalances in long-term ecosystem functioning [2,3]. Soil microorganisms are the dominant organisms in soil and play a crucial role in ecosystem functioning, as they regulate a large proportion of soil processes [4]. Among these microorganisms, soil fungi are particularly significant, serving as decomposers, nutrient cyclers, plant symbionts (e.g., mycorrhizal fungi and rhizobia), and even pathogens, and are therefore central to soil ecosystem functions. In addition, products of arbuscular mycorrhizal fungi (AMF) contribute to the formation and stabilisation of soil aggregates, further underscoring their importance for soil physical structure [5]. Despite the recognised importance of soil fungi, their responses to pesticide application remain poorly understood.

Most studies to date have focused on bacteria or on microbial activities such as respiration and enzyme activities [1,6,7,8,9,10], often under long-term exposure or mediated indirectly through plant responses. Even emerging studies on fungal communities have emphasised chronic or cumulative exposures, with relatively little attention to immediate, direct effects following pesticide application. Yet, short-term perturbations can trigger rapid, differential responses among fungal guilds and other microbial groups, potentially altering ecosystem processes before any overall diversity changes are detectable [11].

For analysing overall community complexity, the use of traditional diversity metrics (e.g., species richness, Shannon index, or evenness) remains essential in microbial ecology studies. Though these indices help capture shifts in community structure, such as the total number of taxa, their evenness, and dominance under pesticide pressure, they may overlook disruptions to important functional roles within fungal assemblages. Specifically, two fungal communities can exhibit similar diversity values yet differ in the ecological functions they perform due to distinct trophic strategies, such as mycorrhizal associations with symbionts, decomposition by saprotrophs, or pathogenic influence by pathogens. Thus, integrating trophic group analysis offers a more nuanced assessment of pesticide impacts, capturing changes in functional composition and potential disruptions to nutrient cycling, carbon turnover, and plant-fungi interactions long before overall diversity is affected [12,13].

Recent studies have demonstrated that pesticide applications can differentially alter taxonomic and functional aspects of soil microbial communities, with some taxa and trophic groups being more strongly affected than others, including notable changes in fungal community structure and microbial network interactions [14,15]. While most of these studies focus on long-term or indirect effects mediated by plants or soil processes, evidence indicates that even short-term, direct applications of pesticides can cause rapid shifts in community composition, especially among fungi. For instance, abrupt application of both herbicides and fungicides has been shown to modify fungal community structure within weeks, although the sensitivity of specific fungal guilds remains to be fully elucidated [11]. This highlights the importance of distinguishing between direct toxic effects, such as membrane or enzyme inhibition caused by fungicides, and indirect effects mediated via changes in plant–soil interactions, which often predominate in herbicide studies.

Herbicides and fungicides differ in their modes of action and expected impact on soil fungi: fungicides exert direct toxic effects, while herbicides primarily affect fungi indirectly via alterations in plant-derived substrates or root exudates. However, a substantial proportion of many herbicide formulations enters the soil directly after application [16], where they can exert additional direct effects on microbial cells, depending on their chemical properties, sorption behaviour, and degradation dynamics. Studying both pesticide types in parallel provides a comprehensive understanding of their immediate influence on fungal communities.

The microbial community’s response to pesticide exposure is also affected by various environmental factors, such as pesticide concentration and combinations, application methods, soil characteristics (including soil type, pH, organic matter, land use history, etc.), and the composition of the resident microbial community [17,18,19,20,21,22]. The soil environment influences pesticide mobility and degradation rates, ultimately controlling biological exposure [23,24]. Among these factors, soil pH plays a major role in shaping microbial diversity and community assembly [25,26]. Each pesticide varies in chemical structure, mode of action, and persistence, which determines its selective impact on microbial groups [7,27]. As a result, the same pesticide can cause different microbial responses depending on the soil type or management practices [18,28].

Given these gaps, particularly the limited understanding of short-term direct effects of herbicides and fungicides on soil fungi, and the ecological importance of early responses including mycorrhizal and rhizobial interactions, our study aims to assess the immediate direct effects of different pesticide applications across three different soils on α- and β-diversity, taxonomic and trophic-group compositions, and to identify taxa-specific associations with pesticide exposure. Specifically, we hypothesised that (H1) short-term pesticide exposure would reduce soil fungal diversity and consistently alter fungal community composition across soils, with trophic groups differing in their sensitivity to pesticide-induced disturbance regardless of the soil type or soil pH, and (H2) fungicides exert a stronger negative effect than herbicides due to their direct mode of action.

## 2. Materials and Methods

### 2.1. Study Description

The study was conducted as a pot experiment following a fully factorial design with two factors: pesticide treatment and soil type.

#### 2.1.1. Pesticides and Soil Selection

We used eight commercially available pesticides, comprising three fungicides: Mirador XTRA, Kuprikol 50, and Captan 80 WG, and five herbicides: Basagran, CORUM, Stomp 400 SC, Targa Super 5 EC, and Sharpen 40 SC. The selection focused on pesticides commonly used to protect legumes. All pesticides were applied and evaluated as commercial formulations rather than as isolated active ingredients, reflecting their practical use in agricultural management.

To assess the effects of these pesticides on soil fungal communities, we collected three soils from ecologically managed agricultural fields that differed in pH (Table 1) and had been used in a previous study [1]. The selected fields had been pesticide-free for approximately 10 years, as documented in a previous study [1].

Soil physicochemical characteristics, available phosphorus (P), potassium (K), magnesium (Mg), calcium (Ca), and nitrogen forms; nitrate (NO_3_^−^) and ammonium (NH_4_^+^), were determined in the air-dried and homogenized soil samples before experiment establishment across all three soil types, following Řezáčová et al. (2021) [29] (Table 1). The Mehlich III extraction method [30] was used for measuring the available P, K, Mg, and Ca, and quantified with an Agilent ICP-OES 5110 VDV instrument (Agilent Technologies, Santa Clara, CA, USA). Nitrate (NO_3_^−^). The level of NH_4_^+^ was determined according to ISO 14255:1998 [31] using calcium chloride as the extractant and analyzed with an automated chemistry analyzer (Skalar Analytical B.V., Breda, The Netherlands). Soil pH at the point of sampling was assessed in a water slurry (1:5, *w*:*v*) following 1 h of shaking and using a pH meter (Hanna Instruments, Woonsocket, RI, USA). Two technical replicate measurements were performed per pot.

Soil physicochemical properties other than pH were used to characterize the three source soils and were not measured independently for each experimental pot. Therefore, these variables were treated as descriptive baseline soil characteristics rather than as independently replicated explanatory variables in subsequent correlation or multivariable analyses.

#### 2.1.2. Pot Experiment

In the pot experiment, one-liter black plastic pots (10 × 10 × 11.5 cm) sterilized with 96% ethanol (Penta, Prague, Czech Republic) were filled with homogenized fresh soil (approximately 1 L) and watered to near field capacity, allowing slight leaching from the bottom, before pesticide application. The pots were randomly arranged within the space to minimize positional effects. Pesticide working solutions were prepared in a final volume of 500 mL according to the manufacturer’s recommended field application rates (Supplementary Table S1). For each pot, an area-adjusted volume corresponding to the 169 cm^2^ soil surface was applied evenly to the soil surface (Supplementary Table S1). Where necessary, the application volume was adjusted with water so that all pesticide-treated and control pots received the same total liquid volume. Control pots received the same total volume of water without pesticide, and no substantial visible drainage was observed after application. The experiment was maintained in a room with an average temperature of 21.4 °C. The experiment was carried out under plant-free conditions.

Four biological replicate pots were established for each combination of soil and pesticide, including control, resulting in a total of 108 pots (3 soils × 9 pesticide treatments × 4 replicates). For each soil type, a control treatment was included and sprayed with the same volume of water as the liquid volume applied for the pesticide-treated pots. The sampling was conducted two weeks after the pesticide application to allow the degradation of DNA from lysed microorganisms in the soil, as microbial DNA typically degrades within hours to a few days in biologically active soils, while only a minor fraction may persist longer when adsorbed to mineral or organic particles [32,33,34]. Soil samples were randomly collected from several points at a depth of 0–10 cm per pot using a sterile metal spoon and pooled to obtain a single composite, homogenized sample per pot.

### 2.2. DNA Isolation and Sequencing

The total genomic DNA was isolated from 0.25 g of fine soil sieved through a 2 mm mesh after air-drying at room temperature, using DNeasy PowerSoil DNA Isolation Kit (QIAGEN, Hilden, Germany) according to the manufacturer’s instructions. The fungal Internal Transcribed Spacer (ITS) region was amplified using the gITS7 (5′-GCGTGARTCATCGARTCTTTG-3′) [35] and ITS4 (5′-ACTCCTCCGCTTATTGATATGC-3′) [36] primers. 5′ end of each forward primer was tagged with 2-nucleotide-long barcodes and the reverse primer with 6-nucleotide-long barcodes. Combi PPP master mix (Top-Bio, Vestec, Czech Republic) was used for the PCR amplification under the thermal cycling conditions with initial denaturation at 94 °C for 2 min, followed by 32 cycles of 30 s denaturation at 94 °C, 30 s annealing at 59 °C, 60 s elongation at 72 °C, and final elongation at 72 °C for 15 min. Amplified PCR products were purified using the Gel/PCR DNA Fragments Extraction Kit (Geneaid Biotech, New Taipei City, Taiwan) according to the manufacturer’s instructions and quantified by a Quantus^TM^ fluorimeter (Promega, Madison, WI, USA) before pooling. An amplicon library was assembled, pooling equimolar concentrations (20 ng) of PCR amplicons from each sample separately for amplicon sequencing. Illumina MiSeq paired-end (2 × 250 bp) sequencing was performed at the company SEQme sro, Dobris, Czech Republic.

### 2.3. Real-Time PCR

Given the key role of AMF in plant–soil interactions and soil ecosystem functioning, we also quantified the total detectable DNA abundance of AMF. To this end, we used NS31 (5′-TTGGAGGGCAAGTCTGGTGCC-3′) [37] and AML2 (5′-GAACCCAAACACTTTGGTTTCC-3′) [38] primers targeting a fragment of the small-subunit ribosomal RNA gene (SSU/18S rRNA) of AMF. Synthesized and HPLC-purified primers were obtained from Generi Biotech (Hradec Králové, Czech Republic). Luna Universal qPCR Master Mix (New England Biolabs, Ipswich, MA, USA) was used for the qPCR, an intercalating-dye-based chemistry; no hydrolysis probe was used. The reaction mixture was prepared according to the master mix manufacturer’s recommendation, and the final volume was adjusted to 20.0 µL with PCR-grade water (PCR Ultra H_2_O, Top-Bio, Vestec, Czech Republic). qPCR reactions were performed on the Light Cycler 480 platform (Roche Diagnostics GmbH, Mannheim, Germany) with the following PCR conditions: initial denaturation for 3 min at 95 °C, followed by 55 cycles of 10 s at 95 °C, 15 s at 60 °C, and 25 s at 72 °C. Melting curve analysis was conducted after each run to assess amplification specificity. The standard curve was generated through serial dilutions of a standard PCR fragment. Three technical replicate measurements were performed for each sample. Copy numbers were calculated using the regression equation (copy number (Cp) = 10^((Cp − intercept)/slope)^, correlating crossing point (Cp) values with known copy numbers from the standard curve. The total DNA abundance of fungi is expressed as copies per gram of dry soil weight.

### 2.4. Sequencing Data Analyses

The raw sequence data were processed using the SEED 2.1.2 pipeline [39]. We merged the FASTQ paired-end reads, quality filtered, and removed shorter sequences (<30 bp). In the next step, the reads were demultiplexed, and the fungal ITSx regions were extracted. Extracted ITSx sequences were clustered into Operational Taxonomic Units (OTUs) using a 97% similarity threshold with the UPARSE pipeline [40] (Edgar, 2013). The reference database, UNITE (10.0 2024-04-04 (all eukaryotes)) [41], was employed for assigning taxonomical information to fungal OTUs. Then, sequencing completeness was evaluated using Good’s coverage (ranged from 97.3% to 99.4% (mean 98.8%)) and sample-based rarefaction curves generated from the quality-filtered OTU table. (Supplementary Table S2 and Supplementary Figure S1). Furthermore, non-fungal data were filtered out, and the OTUs’ abundance in each sample were rarefied to the lowest-count sample (7300 sequences per sample) and then standardized to 100%. We also created a second dataset containing only AMF, as one of the most widespread and ecologically important groups of soil fungi. After removing all other fungal sequences, this dataset was likewise standardized to 100% of the sequences per sample. Finally, alpha diversity for fungi (including AMF; first dataset) and for AMF alone (second dataset) was calculated for each sample using species richness, Shannon diversity H′, and the Buzas–Gibson evenness index (e^H^′/S) in PAST 4.03 [42] software. Taxonomic assignment was performed using the UNITE database within the SEED 2.1.2 pipeline. Trophic groups (e.g., saprotrophs, endophytes, pathogens (see Table S3 for all) were assigned within the SEED 2.1.2 framework based on ecological annotations associated with the identified fungal taxa. No separate external guild-assignment tool, such as FUNGuild or FungalTraits, was used. Of the 9206 fungal OTUs identified, 2928 (31.8%) were could not be assigned to a trophic group and were classified as “Not defined”.

### 2.5. Statistical Data Analysis

RStudio (Open-Source Edition (AGPL v3), statistical software, PAST (5.2.2), or Statistica 14.1.0 (TIBCO Software 224 Inc., Palo Alto, CA, USA) were employed for the statistical data analysis in this study. Statistical significance, alpha was set at 0.05 for the analysis. Before conducting the analysis, data were checked for normality (Shapiro–Wilk test) and equality of variance (Levene’s test). When these assumptions were not met, the variables (i.e., AMF total DNA abundance, fungal taxa richness, community evenness, and Shannon index) were log-transformed before two-way ANOVA to improve normality and homogeneity of variances. Statistical analyses were performed on the transformed values, whereas descriptive statistics and figures are presented using the original indices. We performed two-way ANOVA to assess the influence of pesticide application on AMF total DNA abundance and fungal diversity metrics. Tukey’s HSD tests were performed for post-hoc comparisons. To evaluate the pesticide treatment-specific effects on microbial trophic groups, we conducted pairwise comparisons between each pesticide treatment and the control using the non-parametric Wilcoxon rank-sum tests. The results were interpreted cautiously to avoid the risk of false positives associated with multiple pairwise comparisons.

We further conducted permutational multivariate analysis of variance (PERMANOVA) on Bray–Curtis dissimilarities using 999 permutations, adonis2, to evaluate the effect of pesticide application on fungal community composition in each soil type. Dispersion was tested to confirm the variation caused by the pesticide treatments. We focused on the influence of each pesticide individually and the pairwise comparison of each pesticide treatment with the control treatment. To visualize the fungal community shift under the influence of pesticide treatments in each soil, Principal Coordinate Analysis (PCoA) was carried out for each soil separately.

To identify pesticide-specific responses on soil pH and treatments contributing to the overall community composition, each treatment was compared against the combined set of all remaining treatments using Welch’s *t*-test and PERMANOVA, respectively. The Pearson correlation was conducted to determine the correlation between total AMF DNA abundance and soil pH. In addition, PERMANOVA test was conducted to determine the influence of pH on fungal community composition and to monitor the indirect influence of pesticides on community composition through altering soil pH. For the same reason, Ordinary Least Squares Regression (Pearson correlation) analysis was used to assess the potential correlations between fungal diversity metrics and soil pH.

Additionally, we applied two complementary approaches to identify individual fungal taxa associated with pesticide treatments. First, to test the strength of the association of each taxon with individual pesticide treatments, based on their specificity and consistency of occurrence, we performed Indicator species analysis (IndVal). Redundancy analysis (RDA) followed by t-value biplots was conducted (using CANOCO 5.10 software) to assess the statistical significance and direction of taxon responses along the pesticide application. This allowed us to understand the taxa whose abundance significantly altered along the explanatory variable, regardless of exclusiveness.

## 3. Results

### 3.1. Influence of Pesticides on Soil Fungal Diversity and Community Composition

**Figure 1:**
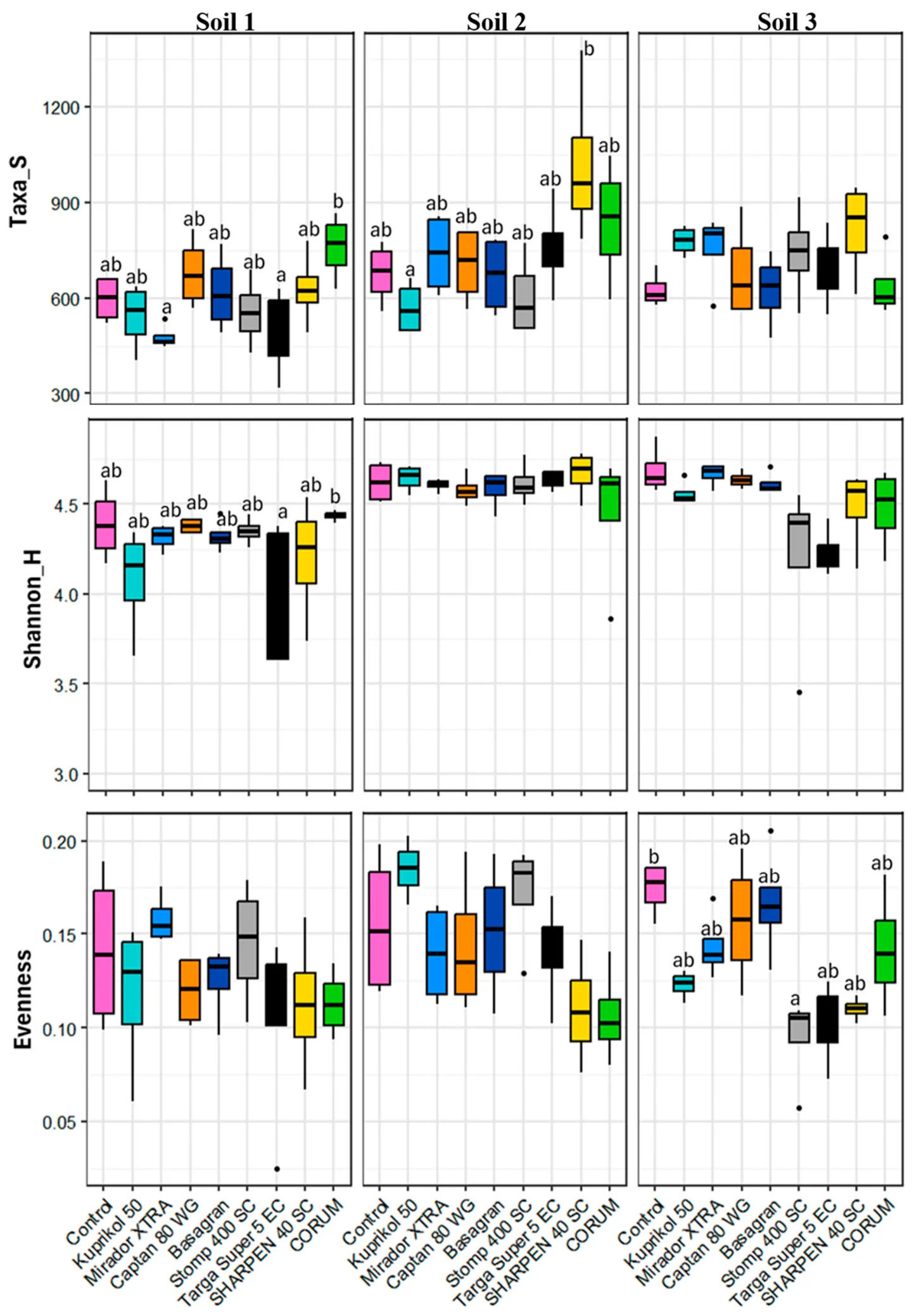
Fungal diversity: taxa richness (Taxa_S), Shannon diversity index (Shannon_H), and community evenness (Evenness) under different pesticide treatments in different soil types. Different lowercase letters indicate significant differences in the means of fungal diversity at α = 0.05, as determined by Tukey’s HSD test, highlighting within-soil variability in response to treatment.

**Figure 2:**
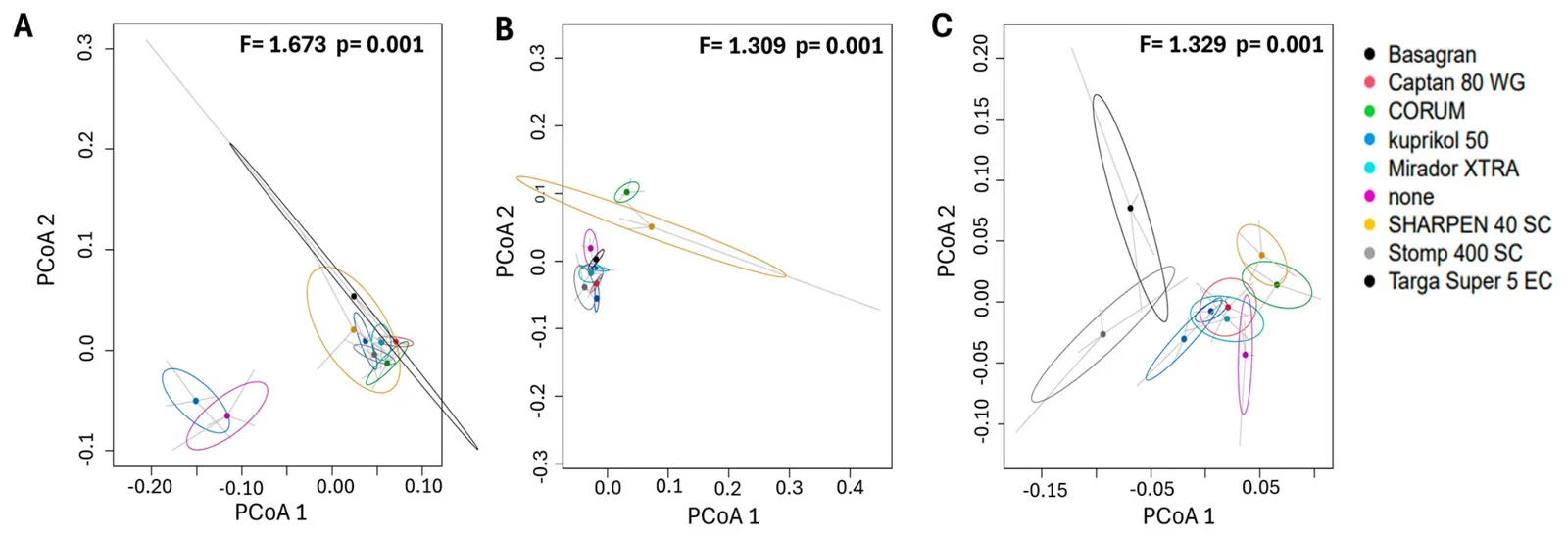
Principal Coordinates Analysis (PCoA) plot showing the fungal community compositions across pesticide treatments in three soil types: (**A**) Soil 1, (**B**) Soil 2, and (**C**) Soil 3, based on Bray–Curtis dissimilarity. Each ellipse indicates a 95% confidence interval around the group centroid. Axes represent the first two principal coordinates (PCoA1 and PCoA2), summarizing the major variation in community composition. Lines connect individual samples to their group centroid, illustrating within-group variation (beta dispersion). Dispersion was assessed using the *betadisper()* function to test for homogeneity of group variances among distance treatments, followed by a permutation test. Treatment “none” represents the control treatment.

Overall, alpha diversity and community composition of fungi showed significant pesticide- and soil-type-dependent responses (Table 2 and Table 3, Figure 1 and Figure 2). The two-way ANOVA indicated significant effects of soil type, pesticide treatment, and their interaction for Taxa richness, while the Shannon diversity index and Evenness showed weaker and less consistent responses (Table 2). However, post hoc comparisons within individual soils did not identify any individual pesticide treatment that significantly differed from the control in Taxa richness and Shannon diversity index (Figure 1). In contrast, fungal community composition responded more clearly to pesticide application, particularly in Soil 1 (Table 4, Figure 2A).

Pesticide-induced changes in fungal communities were detected at the level of community composition, with certain treatments differing from the control (Table 3, Figure 2).

Community compositions of fungi differed from the control in response to all tested pesticides in soil 1 (Table 4, Figure 2A). In soil 2, only communities under CORUM and SHARPEN 40 SC, and in soil 3 under Kupricol 50, Stomp 400 SC, SHARPEN 40 SC, and Targa Super 5 EC differed from controls (Table 4, Figure 2B and Figure 2C, respectively).

### 3.2. Influence of Pesticides on Soil Fungal Trophic Groups

**Figure 3:**
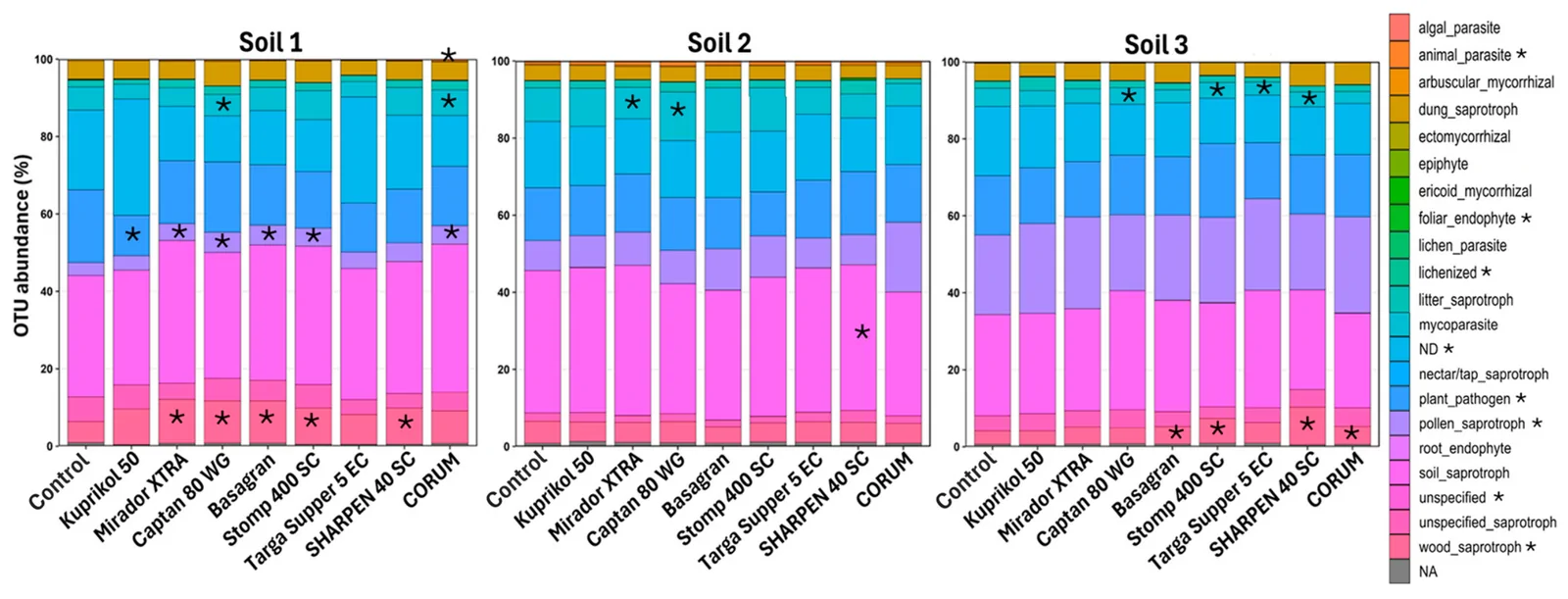
Pesticide-driven shifts in the relative abundance (%) of fungal trophic groups across three soils under different pesticide treatments. Asterisks indicate treatments with statistically significant (at α = 0.05) effects compared to the control within each soil.

Pesticide treatments had limited, soil-, and pesticide-dependent effects on the relative abundance of fungal trophic groups (Table 5, Figure 3). Compared to control, some pesticides affected only a limited number of trophic groups, whereas others generated broader responses across multiple groups (Table 5). Kuprikol 50 and Targa Super 5 EC exhibited the weakest effects, each significantly influencing only a single trophic group, whereas Stomp 400 SC showed the strongest response, affecting five trophic groups across soils. The abundance of other saprotrophic, parasitic, epiphytic, and symbiotic fungi, including the key soil fungi, AMF, were not affected by pesticide treatment.

Although pesticide effects on trophic-group relative abundances were generally limited, overall, community composition within trophic groups was highly responsive to pesticide treatments (Supplementary Table S3). Most individual pesticides showed a significant shift in community composition relative to the control across a wide range of trophic groups. AMF, foliar endophytes, pollen saprotrophs, wood saprotrophs, plant pathogens, and mycoparasites showed significant community shifts across all pesticide treatments. In contrast, moss symbionts, unspecified pathotrophs, and sooty mold exhibited no compositional changes under any pesticide treatment (Supplementary Table S4).

#### Variation in Total Abundance of Soil AMF Under the Influence of Pesticides

**Figure 4:**
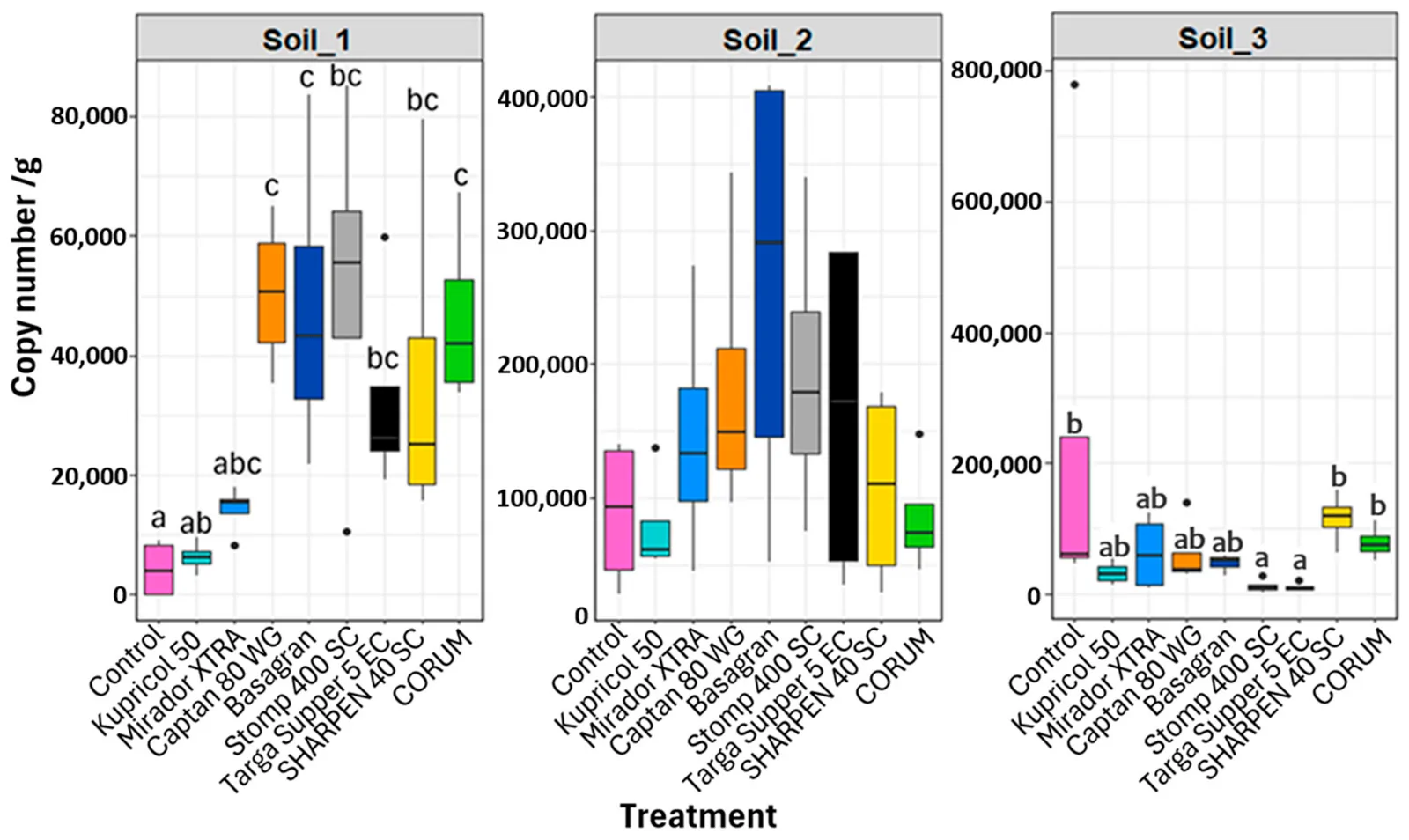
Total DNA abundance of arbuscular mycorrhizal fungi (AMF) under different pesticide treatments in three different soil types (Soil 1, Soil 2, and Soil 3) compared to the control treatment (none). Different lowercase letters indicate significant differences among pesticide treatments within each soil type at a significance level α = 0.05, based on the Tukey HSD test. No letters are shown for Soil 2 because no significant differences among treatments were detected within this soil.

We found a significant influence of pesticides on total DNA abundance (qPCR copy numbers) of AMF (F_8,79_ = 3.91, *p* = 2.4 × 10^−4^), based on two-way ANOVA, and a significant soil type-pesticide interaction (F_16,79_ = 4.24, *p* = 7.55 × 10^−6^) (Figure 4). Subsequent Tukey’s HSD post hoc comparisons were performed within each soil type. In soil 1, all pesticides, except for Kuprikol 50 and Mirador XTRA, which had no effect, increased total AMF DNA abundance compared to the control. In contrast, in soil 3, Targa Super 5 EC and Stomp 400 SC decreased total AMF DNA abundance, while the other pesticides had no effect. In soil 2, there were no significant differences in total AMF DNA abundance among pesticides (Figure 4).

### 3.3. Individual Taxa Significantly Associated with Pesticide Treatments

To identify individual taxa significantly associated with different pesticides. RDA-based t-value biplots with van Dobben circles revealed a set of individual taxa significantly associated with pesticide treatments (Supplementary Figure S2). The associations are usually both positive and negative. Only pesticide SHARPEN 40 SC appeared to have a negative impact on individual taxa in soil 2.

### 3.4. Association of Soil pH with Fungal Diversity Metrics, Community Compositions, and Total Abundance of AM Fungi

Fungal diversity, including taxa richness (r = −0.3769, *p* = 5.81 × 10^−5^), Shannon index (r = −0.4657, *p* = 3.80 × 10^−7^), and evenness (r = −0.4792, *p* = 1.55 × 10^−5^), showed significant negative correlation with soil pH. Moreover, the soil pH showed a significant effect on fungal (F = 41.613, *p* = 1.0 × 10^−3^) community composition across soils, explaining 28.2% of observed variation in community structure. Additionally, the total AMF abundance showed a significant negative correlation with soil pH (r = −0.4648, *p* = 5.19 × 10^−7^).

Comparison of individual treatments against all remaining treatments combined revealed that the changes in soil pH and fungal community composition associated with individual pesticides were not always consistent in response following pesticide application (Supplementary Table S5). For example, Mirador XTRA significantly altered soil pH in Soil 1 (df = 4.453, *p* = 0.048) without affecting fungal community composition (pseudo-F = 0.735, *p* = 0.431), whereas Stomp 400 SC significantly affected fungal community composition in Soil 3 (pseudo-F = 2.777, *p* = 0.013) while having no significant effect on soil pH (df = 32.167, *p* = 0.052).

## 4. Discussion

Our results show that short-term direct pesticide exposure primarily reshaped soil fungal communities in a soil-dependent manner, with community composition responding more strongly and consistently than alpha diversity or trophic-group abundance. Across the three agricultural soils, pesticide effects were selective rather than uniformly suppressive. These findings indicate that short-term pesticide exposure can rapidly reorganise fungal communities without consistent losses in diversity depending on the soil context.

### 4.1. Effect of Pesticides on Fungal Diversity and Community Composition

Fungal responses to pesticide exposure were strongly context-dependent, with both the magnitude and direction of the response varying among soils and individual treatments. This was most evident in the contrasting behavior of the three soils: Soil 1 showed the strongest and most widespread responses, Soil 2 remained comparatively resistant, and Soil 3 exhibited intermediate but treatment-specific shifts.

Such variability suggests that fungal responses were shaped not by a single uniform mechanism, but by interactions among pesticide-specific properties, soil physicochemical characteristics, resident community composition, and pesticide bioavailability. Similar context-dependent fungal responses to pesticide exposure have been reported previously [43,44].

Although pairwise post-hoc comparisons did not reveal significant differences in alpha diversity relative to the control, some treatments, such as Sharpen 40 SC in Soil 2, showed numerical increases that may point to differential responses among fungal taxa. In our study, however, these patterns were more clearly reflected in community composition than in alpha-diversity metrics, suggesting that short-term pesticide exposure may initially induce taxonomic reorganization before corresponding changes become detectable in richness or evenness.

Furthermore, our results highlight the importance of considering commercial formulations when assessing pesticide effects on fungal communities. In our study, pesticides were evaluated as marketed products rather than as isolated active ingredients. Therefore, differences between products sharing the same active ingredient, such as Stomp 400 SC and Sharpen 40 SC, or products partially overlapping in active-ingredient composition, such as Basagran and CORUM, may reflect not only the shared active ingredient, but also formulation composition, co-formulants, additional active ingredients, and differences in active ingredient load.

### 4.2. Influence on Trophic Groups

We observed highly soil- and pesticide-specific responses of fungal trophic groups, with some groups showing significant changes in relative abundance, whereas others remained stable. Importantly, pesticide exposure altered community composition within trophic groups across a much broader range of groups than was apparent from abundance data alone.

This pattern is important because changes in the taxonomic composition of trophic groups may translate into altered ecosystem functioning, even in the absence of strong quantitative shifts at the group level. Different taxa assigned to the same trophic category can differ in their ecological traits, including substrate use, environmental tolerance, and their contribution to nutrient cycling or plant health [45,46].

Consequently, pesticide-driven turnover within saprotrophic, pathogenic, symbiotic, or parasitic groups could modify decomposition processes, plant-fungus interactions, or antagonistic relationships in soil without being captured by trophic-group abundance alone. At the same time, our short-term design does not allow direct inference about realised functional change, so these results are best interpreted as evidence of functional reorganization potential rather than demonstrated functional loss or gain.

### 4.3. Soil-Dependent Fungal Responses and the Role of pH

The contrasting responses among the three soils suggest that pesticide effects were partly mediated by soil-specific conditions, among which pH appears to have played an important role. In our dataset, pH was the only measured parameter that both responded to pesticide treatment (Supplementary Table S5) and was significantly associated with fungal diversity, community composition, and AMF total abundance. This indicates that pesticide-induced shifts in pH may have contributed to the observed fungal responses, at least in some soil-treatment combinations. However, these relationships were not consistent across all treatments: some pesticides altered fungal community composition without detectable pH change, whereas others affected pH without a corresponding compositional response. Thus, pH likely represents one important pathway through which pesticides influence soil fungi, but it does not fully explain the soil-dependent variability observed in this study.

The same soil context was also reflected in AMF total fungal abundance, which increased under several pesticide treatments in Soil 1, decreased under selected treatments in Soil 3, and remained unchanged in Soil 2. These contrasting responses suggest that the same pesticide can stimulate, suppress, or leave AMF abundance unaffected depending on the ecological context of the soil. The apparent resistance of Soil 2 may therefore reflect a greater buffering capacity or lower biological exposure to pesticides, although the specific mechanisms remain unresolved. Together, these findings indicate that pH contributed to the observed responses, but acted alongside other soil-specific characteristics, including the initial structure of fungal communities and broader physicochemical conditions.

However, these findings cannot be interpreted as evidence of AMF growth. Arbuscular mycorrhizal fungi are obligate biotrophs that require living host roots to complete their life cycle, and active biomass production is expected to be limited in plant-free systems [47,48]. Therefore, the observed changes in qPCR copy number likely reflect changes in AMF DNA abundance associated with existing propagules or hyphae, DNA persistence in soil or other limitations in DNA-based quantification in host-free systems, rather than fungal growth.

### 4.4. Evaluation of Hypotheses

Taken together, our results provided only partial support for the proposed hypotheses. H1 predicted that short-term pesticide exposure would reduce fungal diversity and consistently alter fungal community composition across soils, with trophic groups differing in their sensitivity to pesticide-induced disturbance irrespective of soil context. This hypothesis was only partially supported. Fungal diversity was not consistently reduced relative to the control, and pesticide effects were strongly soil- and treatment-dependent rather than uniform across soils. However, H1 was supported with respect to community composition, which responded more clearly than alpha diversity, and with respect to trophic groups, which differed in their sensitivity to pesticide exposure. These differences were expressed more clearly through changes in internal community composition than through consistent shifts in group-level abundance.

H2 was also only partially supported: although stronger negative effects of fungicides were expected due to their direct mode of action, their effects were not consistently stronger than those of herbicides across soils and response variables.

### 4.5. Study Limitations

Several limitations of this study should be considered when interpreting the results. First, our experiment captured only short-term responses two weeks after pesticide application and therefore does not resolve whether the observed community shifts persist, intensify, or recover over time. At the same time, this short-term design is also a strength, because it specifically captures early direct responses of soil fungi before longer-term recovery, indirect plant-mediated pathways, or cumulative field effects become dominant. Second, the pot-based design allowed us to isolate direct soil effects of pesticides under controlled conditions, but it does not fully reflect field settings, where fluctuating microclimate, repeated applications, plant-mediated interactions, and rhizosphere processes may substantially modify fungal responses. This is particularly relevant for herbicides, whose impacts on soil microbial communities in agricultural systems are often partly mediated through vegetation changes and associated rhizosphere processes. In addition, the absence of plants should be viewed both as a limitation and an advantage: although it reduces field realism, it allowed us to distinguish direct soil-borne pesticide effects from indirect responses mediated through plant performance or root-associated processes. Also, only a limited set of soil variables was measured, so the mechanistic role of pH could be evaluated only partially, and other unmeasured soil properties, such as nutrient availability, organic matter content, texture, moisture, and other drivers of pesticide bioavailability or microbial resistance remain unresolved.

Another limitation of this study is that the DNA-based methods used here do not distinguish viable fungi from extracellular or relict DNA derived from dead or damaged organisms. Although sampling two weeks after application was expected to capture early fungicide responses, DNA released from fungicide-affected cells may still have persisted and remained amplifiable. Consequently, the relatively lower effects of fungicide observed in this study cannot be taken as conclusive evidence of low toxicity, because DNA persistence may have masked reductions in viable or metabolically active fungal populations. This limitation is particularly relevant to the evaluation of Hypothesis 2, which predicted stronger negative effects of fungicides than herbicides.

A further limitation concerns the interpretation of differences between fungicides and herbicides. Because fungicides are designed to target fungi directly, some negative effects on fungal communities are expected a priori and should therefore not be interpreted simply as evidence of greater ecological impact. Instead, the more informative result is that even under these expected differences, responses were not uniformly stronger for all fungicides across all soils and response variables, and several herbicides also induced marked compositional shifts. Finally, amplicon-based community profiling reveals taxonomic restructuring, but does not directly measure realized functional consequences. The observed changes should therefore be interpreted as evidence of altered community structure and functional reorganization potential rather than direct proof of functional loss or gain.

## 5. Conclusions

Overall, our results demonstrate that short-term pesticide exposure reshapes soil fungal communities in a strongly soil-specific, pesticide-dependent, and selective manner. Pesticide effects were expressed primarily through shifts in fungal community composition rather than through consistent reductions in alpha diversity. This indicates that community reorganization can occur rapidly, even when changes in taxa richness, Shannon diversity, or evenness remain limited.

Pesticide effects on fungal trophic groups were also selective. While relative abundances of most trophic groups remained largely stable, community composition within several trophic groups changed markedly, suggesting taxonomic turnover within functional categories rather than broad shifts in trophic-group abundance. In addition, total AMF abundance responded inconsistently among soils, increasing under several pesticide treatments in Soil 1, decreasing under selected treatments in Soil 3, and remaining unchanged in Soil 2.

Collectively, these findings show that pesticide effects on soil fungi cannot be generalized across soils or pesticide types, but depend on interactions among pesticide properties, soil physicochemical characteristics, including pH, and the initial structure of fungal communities. Our results highlight the importance of soil-specific risk assessment that considers fungal diversity and community composition, complemented by quantitative measures such as the abundance of selected fungal groups, including AMF, as indicators of pesticide impacts on soil fungal communities.

## Figures and Tables

**Table 1:** Description of sampling locations and physicochemical profiles of the collected soils.

	Soil_1	Soil_2	Soil_3
Field owner	VH AGROTON, s.r.o., Velké Hostěrádky, Czech Republic	BIOFARMA Sasov, Jihlava, Czech Republic	Ing. Přemysl Čech, Zábřeh Czech Republic
GPS coordinates	49°1′52.43″ N, 16°52′20.64″ E	49° 22′ 51.69″ N, 15° 36′18.014″ E	49°52′59.41″ N, 16°51′50.47″ E
available P (mg/kg)	11.25	111.50	101.60
Mg (mg/kg)	410.00	178.80	114.90
K (mg/kg)	214.00	271.00	280.80
Ca (mg/kg)	7854.00	1863.00	2198.00
Hot water extractable P (mg/kg)	3.29	12.37	11.93
NH_4_ (mg/kg)	6.34	10.15	11.18
NO_3_ (mg/kg)	7.95	13.31	13.95
pH_water_	8.20	6.40	7.00

Sampling site information partly adopted from Némethová et al. (2026) [1].

**Table 2:** The effect of pesticides and soil type on fungal diversity. The table shows the results of the two-way ANOVA statistical analyses. Bolded values indicate statistically significant results (*p* < 0.05).

Diversity	Treatment								
	Soil Type			Pesticide			Soil: Pesticide		
	df	F	*p*	df	F	*p*	df	F	*p*
Taxa richness	2	11.86	**3.03 × 10^−5^**	8	3.25	**2.94 × 10^−3^**	16	2.26	**8.92 × 10^−3^**
Shannon index	2	10.65	**7.82 × 10^−5^**	8	1.63	**1.3 × 10^−2^**	16	1.09	3.76 × 10^−1^
Evenness	2	2.83	**6.51 × 10^−3^**	8	2.78	**9.06 × 10^−3^**	16	1.72	5.98 × 10^−2^

**Table 3:** The influence of pesticides and soil type on fungal community composition. The table shows the results of permutational multivariate analysis of variance (PERMANOVA). PERMANOVA was performed on Bray–Curtis dissimilarities using 999 permutations (adonis2(dissimilarity matrix ~ pesticide + soil type + pesticide: soil type, nperm = 999)). Bolded values indicate statistically significant variables (*p* < 0.05).

Treatment	Statistics						
	PERMANOVA-Bray–Curtis				Dispersion		
	Degree of Freedom	Pseudo-F	R2	*p*	Degree of Freedom	Pseudo-F	*p*
Pesticide	8	1.377	0.052	**0.029**	8	1.087	0.379
Soil type	2	47.084	0.449	**0.001**			
Pesticide: Soil type	16	1.486	0.113	**0.002**			
Soil 1	8	1.673	0.331	**0.001**	8	1.920	0.098
Soil 2	8	1.309	0.280	**0.001**	8	0.615	0.758
Soil 3	8	1.329	0.282	**0.001**	8	1.163	0.356

**Table 4:** Pairwise comparison of the effect of each pesticide on fungal community compositions compared to the control treatments in each soil separately. Pairwise PERMANOVAs were performed on Bray–Curtis dissimilarities using 999 permutations (adonis2(as.dist(distance) ~ treatment, nperm = 999)). Bolded values indicate statistically significant variables (*p* < 0.05).

Treatment	Soil 1		Soil 2		Soil 3	
	Pseudo-F_1.6_	*p*	Pseudo-F_1.6_	*p*	Pseudo-F_1.6_	*p*
Kuprikol 50	1.402	**0.040**	1.165	0.054	1.171	0.050
Mirador XTRA	2.322	**0.033**	1.177	0.067	0.955	0.597
Captan 80 WG	2.671	**0.024**	1.161	0.113	1.136	0.071
Basagran	2.004	**0.029**	1.171	0.056	1.141	0.114
Stomp 400 SC	2.072	**0.030**	1.161	0.067	1.464	**0.022**
Targa Super 5 EC	1.592	**0.032**	0.927	0.678	1.765	**0.025**
SHARPEN 40 SC	1.847	**0.022**	1.559	**0.036**	1.368	**0.025**
CORUM	2.524	**0.030**	1.443	**0.029**	1.088	0.241

**Table 5:** Effect of various pesticides on the relative OTU abundance of fungal trophic groups compared to the control treatment in each soil. The table shows *p*-values from the Wilcoxon test for only those trophic groups that were significantly influenced by at least one pesticide in at least one soil. The significance level was set as α = 0.05, with “ns” representing non-significant results under particular pesticide treatment. Df = 4 per group.

		Pesticide							
	Trophic Group	Kuprikol 50	Mirador XTRA	Captan 80 WG	Basagran	Stomp 400 SC	Targa Super 5 EC	SHARPEN 40 SC	CORUM
Soil 1	Not defined fungi	ns	ns	0.029	ns	ns	ns	ns	0.029
	Animal parasites	ns	ns	ns	ns	ns	ns	ns	0.029
	Foliar endophytes	ns	0.027	ns	ns	0.029	ns	ns	ns
	Plant pathogens	0.029	ns	ns	ns	ns	ns	ns	ns
	Pollen saprotrophs	ns	0.029	0.029	0.029	0.029	ns	ns	0.029
	Wood saprotrophs	ns	0.029	0.029	0.029	0.029	ns	0.029	ns
Soil 2	Not defined fungi	ns	0.029	0.029	ns	ns	ns	ns	ns
Soil 3	Not defined fungi	ns	ns	0.029	ns	0.029	0.029	0.029	ns
	Lichenized fungi	ns	ns	ns	ns	0.029	ns	ns	ns
	Wood saprotrophs	ns	ns	0.029	0.0286	ns	ns	0.029	0.029

## Data Availability

Supplementary Materials associated with this article can be found in the online version of the manuscript. All the raw reads obtained in this study were submitted to the NCBI Sequence Read Archive (SRA) database (Accession Numbers: SAMN46427282).

## Supplementary Materials

The following supporting information can be downloaded at https://www.mdpi.com/article/10.3390/jof12080584/s1.
