# Supplementary material for: Short-Term Pesticide Exposure Reshapes Soil Fungal Communities in a Soil-Dependent Manner
Source: J Fungi (Basel). 2026 Aug 7;12(8):584. doi: 10.3390/jof12080584 (PMC13514313; doi:10.3390/jof12080584)
Supplement: Supplementary file 1 [file jof-12-00584-s001.zip › Supplementary Figures.pdf]

Supplementary Figures

Article title

Short-term pesticide exposure reshapes soil fungal communities in a soil-dependent manner

Veronika Řezáčová1, Oushadee A J Abeyawardana1*, Milan Řezáč1, Ema Némethová1

Affiliation

[1]Czech Agrifood Research Center, Drnovská 507, 161 00, Prague 6, Czech Republic

oushadee.abeyawardana@carc.cz, veronika.rezacova@carc.cz

*Corresponding authors:

Oushadee A J Abeyawardana, Czech Agrifood Research Center, Drnovská 507, 161 00 Prague 6 - Ruzyně, Czech Republic, E-mail: oushadee.abeyawardana@carc.cz, Tel: +420 607060362

VŘ and OA both share the first authorship

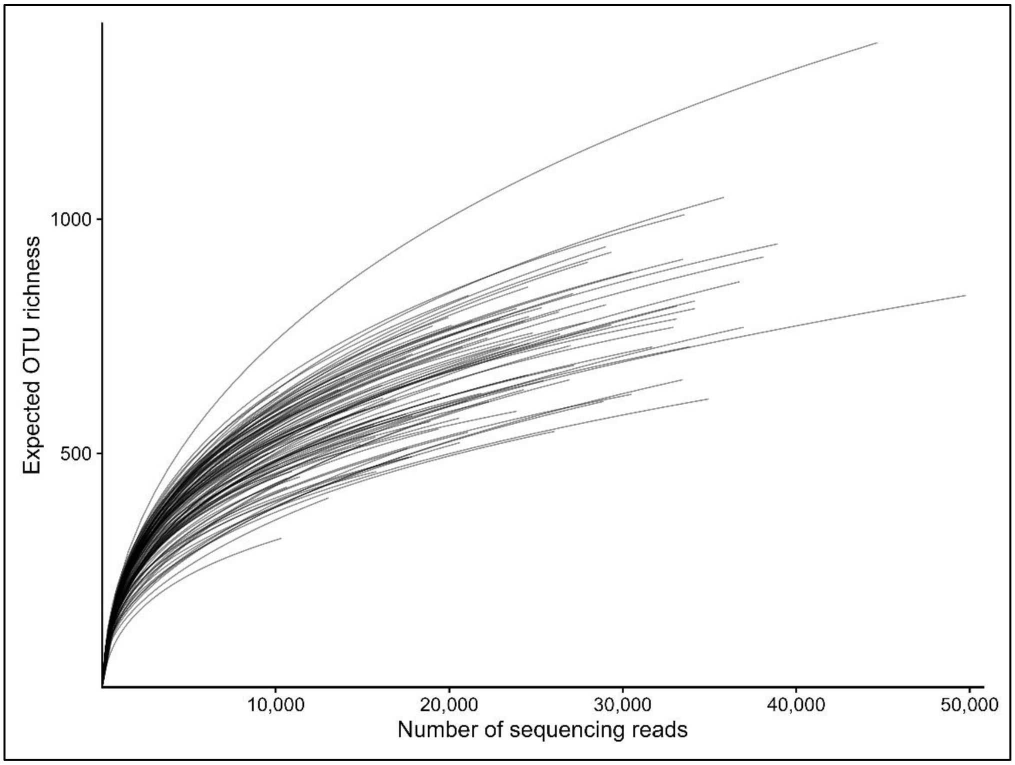

Supplementary Figure S1. Rarefaction curves of fungal OTU richness for individual samples based on Illumina ITS sequencing.

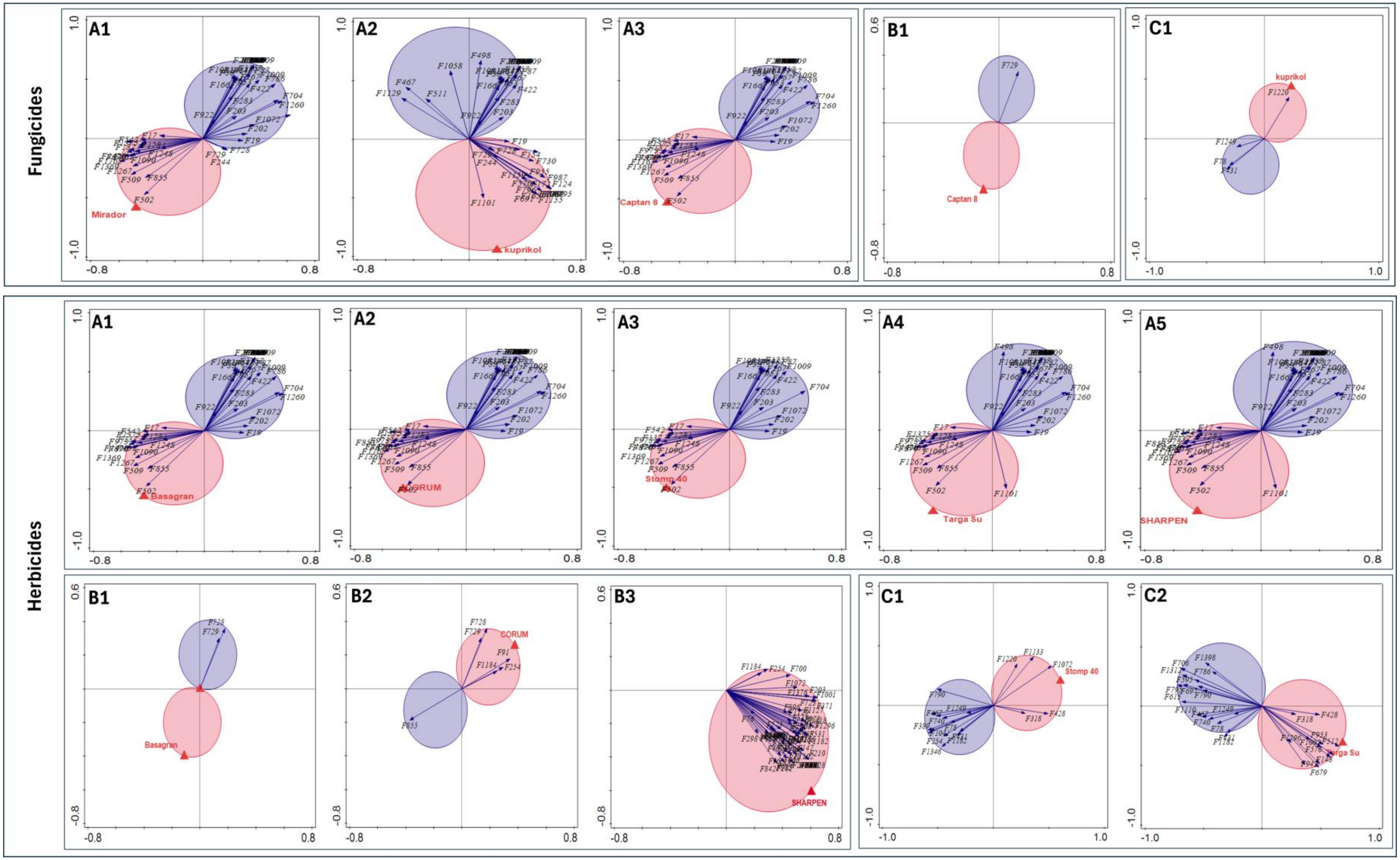

Supplementary Figure S2. T-value biplots for the relationships between individual pesticides and specific soil fungal taxa. The arrows for taxa that fall within Van Dobben's circle indicate a significant relationship (P<0.05) between a particular fungal taxon and a given pesticide. These relationships

can be positive (red circle) or negative (blue circle). Arrows for species outside of Van Dobben's circles have been removed from the graph for clarity. The graph displays only pesticides that showed significant associations with fungal species and fungal species that were significantly positively or significantly negatively associated with the pesticide. The species label consists of a number followed by the abbreviation of a species name. Supplementary Table S6 lists the taxa associated with each pesticide treatment, and Supplementary Table S7 lists the full name of the taxa present by each taxa ID in the figure. A1-A8, Soil 1, B1- B4, Soil 2 and C1 -C3, Soil 3.
