# Supplementary material for: Short-Term Pesticide Exposure Reshapes Soil Fungal Communities in a Soil-Dependent Manner
Source: J Fungi (Basel). 2026 Aug 7;12(8):584. doi: 10.3390/jof12080584 (PMC13514313; doi:10.3390/jof12080584)
Supplement: Supplementary file 1 [file jof-12-00584-s001.zip › Supplementary Tables.pdf]

Article title

Short-term pesticide exposure reshapes soil fungal communities in a soil-dependent manner

Veronika Řezáčová1, Oushadee A J Abeyawardana1*, Milan Řezáč1, Ema Némethová1

Affiliation

[1]Czech Agrifood Research Center, Drnovská 507, 161 00, Prague 6, Czech Republic

oushadee.abeyawardana@carc.cz, eronika.rezacova@carc.cz

*Corresponding authors:

Oushadee A J Abeyawardana, Czech Agrifood Research Center, Drnovská 507, 161 00 Prague 6 - Ruzyně, Czech Republic, E-mail: oushadee.abeyawardana@carc.cz, Tel: +420 607060362

VŘ and OA both share the first authorship

Supplementary Table S1. Details of selected pesticides and their use for field applications, including recommended doses/dilutions.

	Formulation	Manufacturer	Active compound	Dose used [L/ha]	Recommended dose range [L/ha]	Dilution [L/ha]	Recommended dilution [L/ha]	Active compound dose per pot [µL]
Herbicides	Basagran	BASF AG, Agricultural Products	bentazone	2	2	200	200-400	3.38
	Stomp 400 SC	BASF AG, Agricultural Products	pendimethalin	4.1	4.1	400	400-600	6.93
	CORUM	BASF AG, Agricultural Products	imazamox, bentazone	1.25	1.25	100	100-400	2.11
	Targa Super 5 EC	Nissan Chemical Ind. Ltd.	quizalofop-P-ethyl	2.5	1.5-2.5	200	200-400	4.23
	Sharpen 40 SC	Sharda Worldwide Exports Pvt.Ltd.	pendimethalin	4.1	4.1	400	400-600	6.93
Fungicide	Mirador XTRA	Adama CZ s.r.o.	azoxystrobin, cyproconazole	1	1	200	200-400	1.69
	Kuprikol 50	NeraAgro,spol. s r.o.	copper oxychloride	5	5	200	200	8.45
	Captan 80 WG	Arysta LifeScience S.A.S	captan	2.1	2.1	500	500	3.55

Table partly adopted from Némethová et al., (2026).

Supplementary Table S2. Good's coverage values for individual samples and summary statistics, demonstrating high sequencing completeness across the dataset.

	Goods_coverage (%)
Minimum	97.3
First quartile	98.7
Median	98.9
Mean	98.8
Third quartile	99.1
Maximum	99.4

Supplementary Table S3. Effect of pesticide treatments on community composition within each fungal trophic group across all soil types. Analyses were based on Bray–Curtis dissimilarities calculated from OTU-level relative abundance data. Pesticide was included as a fixed factor, and significance was assessed using 999 permutations and α = 0.05. Bolded values indicate statistically significant variables.

Trophic Group	Df	R^2	F	p
Soil saprotrophs	10	0.457412	8.177292	**0.001**
Lichenized fungi	10	0.209852	2.523059	**0.001**
Animal parasites	10	0.337449	4.940384	**0.001**
Wood saprotrophs	10	0.707147	23.42242	**0.001**
Unspecified saprotrophs	10	0.699871	22.61946	**0.001**
Plant pathogens	10	0.531964	11.02491	**0.001**
Foliar endophytes	10	0.779632	34.31728	**0.001**
Litter saprotrophs	10	0.498545	9.643698	**0.001**
Ectomycorrhizal fungi	10	0.160783	1.724284	**0.004**
Arbuscular mycorrhizal fungi	10	0.328225	4.739358	**0.001**
Mycoparasites	10	0.573844	13.06164	**0.001**
Not defined fungi	10	0.429253	7.295283	**0.001**
Dung saprotrophs	10	0.665811	19.32547	**0.001**
Lichen parasites	10	0.247175	2.331146	**0.002**
Sooty molds	9	0.692903	1.754902	0.217
Nectar/tap saprotrophs	10	0.208485	0.948239	0.562
Pollen saprotrophs	10	0.565509	12.62498	**0.001**
Epiphytes	10	0.118785	0.903138	0.605
Root endophytes	10	0.110715	0.746996	0.679
Unspecified pathotrophs	8	0.505983	0.896194	0.564
Algal parasites	10	0.118022	0.89656	0.559
Unspecified fungi	10	0.568958	10.0317	**0.001**

Supplementary Table S4. Effects of individual pesticide treatments on community composition within fungal trophic groups relative to the control treatment across all soils. This table is provided as **a separate Excel file** in the Supplementary Materials. The table shows the results of permutational multivariate analysis of variance (PERMANOVA). PERMANOVA was performed on Bray–Curtis dissimilarities using 999 permutations. Bolded values indicate statistically significant variables ($p < 0.05$).

Supplementary Table S5: Influence of each pesticide treatment relative to the pooled responses of all remaining treatments within each soil type. This analysis was performed to identify pesticides producing responses that differed from the overall distribution of the remaining pesticide treatments rather than to compare treatments with the untreated control. Soil pH differences were evaluated using Welch's two-sample t-test, and fungal community composition was assessed using PERMANOVA based on Bray–Curtis dissimilarities (999 permutations). Significant effects are shown in bold ($p < 0.05$).

	**Soil 1**					**Soil 2**					**Soil 3**				
	**pH**		**Community composition**			**pH**		**Community composition**			**pH**		**Community composition**		
**Pesticide**	**Degree of freedom**	**p-value**	**F**	**R²**	**p-value**	**Degree of freedom**	**p-value**	**F**	**R²**	**p-value**	**Degree of freedom**	**p-value**	**F**	**R²**	**p-value**
**Control**	7.3987	5.67E-02	1.8202	0.051	0.109	20.0840	**1.52E-02**	0.3753	0.011	0.877	4.5826	1.35E-01	1.3895	0.039	0.201
**Mirador XTRA**	4.4525	**4.76E-02**	0.7349	0.021	0.431	4.4586	5.60E-01	0.515	0.015	0.687	3.2700	5.57E-01	0.6817	0.020	0.676
**Captan 80 WG**	6.8134	7.31E-02	1.3064	0.037	0.134	24.7110	6.40E-01	1.0648	0.030	0.306	33.3360	**1.03E-03**	0.7908	0.023	0.557
**Basagran**	4.8511	**4.50E-03**	0.5902	0.017	0.572	7.9927	7.35E-01	0.8987	0.026	0.370	33.9340	4.40E-01	0.4219	0.012	0.887
**Stomp 400 SC**	12.4480	**4.74E-05**	0.6822	0.020	0.452	22.4760	**1.25E-02**	1.2085	0.034	0.256	32.1670	5.18E-01	2.7773	0.076	**0.013**
**Targa Super 5 EC**	6.7769	8.12E-01	2.4364	0.067	0.078	3.1607	1.84E-01	0.6426	0.019	0.563	32.6340	**2.97E-02**	1.421	0.040	0.199
**SHARPEN 40 SC**	18.5580	1.66E-01	0.3349	0.010	0.846	10.1500	**2.71E-04**	2.1642	0.060	0.128	3.3343	5.06E-01	2.1334	0.059	0.059
**CORUM**	4.0365	5.99E-01	0.85	0.024	0.318	5.2260	**7.72E-03**	2.8702	0.078	**0.038**	26.0490	**2.46E-03**	0.9132	0.026	0.482
**Kuprikol 50**	24.4050	**1.51E-06**	3.2718	0.088	0.073	30.3320	**1.03E-02**	0.1776	0.005	0.987	10.4330	**2.48E-06**	1.6031	0.045	0.144

Supplementary Table S6. Fungal taxa showing significant associations with individual pesticide treatments identified by redundancy analysis (RDA) and visualized in the corresponding T-value biplots (Figure 4). This table is provided as **a separate Excel file** in the Supplementary Materials.

Supplementary Table S7. Species listed in t-value biplot. This table is provided as **a separate Excel file** in the Supplementary Materials.
